# Research on the Effects of the Relationship between Agronomic Traits and Dwarfing Genes on Yield in Colored Wheat

**DOI:** 10.3390/genes15060649

**Published:** 2024-05-21

**Authors:** Wurijimusi Li, Xinmei Gao, Geqi Qi, Wurilige, Longyu Guo, Mingwei Zhang, Ying Fu, Yingjie Wang, Jingyu Wang, Ying Wang, Fengting Yang, Qianhui Gao, Yongyi Fan, Li Wen, Fengjiao Li, Xiuyan Bai, Yue Zhao, Bayarmaa Gun-Aajav, Xingjian Xu

**Affiliations:** 1 Department of Biology, School of Arts and Sciences, National University of Mongolia, Ulaanbaatar 14201, Mongolia; jimusi1117@163.com; 2 Hinggan League Institute of Agricultural and Animal Husbandry Sciences, Hinggan League 137400, China; mnygxm@163.com (X.G.); q123456@163.com (G.Q.); wurilige1025@163.com (W.); guolongyu2020@163.com (L.G.); zhangmw2024@163.com (M.Z.); hfuyingimu2013@163.com (Y.F.); 15048238063@163.com (Y.W.); wangboning9@163.com (J.W.); wang1@163.com (Y.W.); yangft2024@163.com (F.Y.); wl123@163.com (L.W.); momofengjiao@163.com (F.L.); bai123@163.com (X.B.); zy123456@163.com (Y.Z.); 3 Hinggan League Agricultural and Animal Husbandry Technology Extension Center, Hinggan League 137400, China; mn1122@163.com; 4 Hinggan League Academy of Occupation and Technology, Hinggan League 137400, China; fyy123@126.com

**Keywords:** colored wheat, agronomic traits, structural equation model, entropy method, dwarfing genes

## Abstract

This research focuses on 72 approved varieties of colored wheat from different provinces in China. Utilizing coefficients of variation, structural equation models, and correlation analyses, six agronomic traits of colored wheat were comprehensively evaluated, followed by further research on different dwarfing genes in colored wheat. Using the entropy method revealed that among the 72 colored wheat varieties, 10 were suitable for cultivation. Variety 70 was the top-performing variety, with a comprehensive index of 87.15%. In the final established structural equation model, each agronomic trait exhibited a positive direct effect on yield. Notably, plant height, spike length, and flag leaf width had significant impacts on yield, with path coefficients of 0.55, 0.40, and 0.27. Transcriptome analysis and real-time fluorescence quantitative polymerase chain reaction (RT-qPCR) validation were used to identify three dwarfing genes controlling plant height: *Rht1*, *Rht-D1*, and *Rht8*. Subsequent RT-qPCR validation clustering heatmap results indicated that *Rht-D1* gene expression increased with the growth of per-acre yield. *Rht8* belongs to the semi-dwarf gene category and has a significant positive effect on grain yield. However, the impact of *Rht1*, as a dwarfing gene, on agronomic traits varies. These research findings provide crucial references for the breeding of new varieties.

## 1. Introduction

Colored wheat is a valuable genetic resource with distinctive grain colors such as black, blue, purple, and green. Due to the abundance of natural pigments in its grains, colored wheat has drawn attention for its unique quality characteristics and development potential [1,2]. Colored wheat can play a significant role in the prevention of various diseases associated with oxidative stress [3,4,5]. However, colored wheat germplasm resources, especially those with both optimal quality and yield-related traits and availability for large-scale planting, are still lacking [6]. Thus, breeding colored wheat with both superior quality and superior yield features has become a popular objective to increase the value of processed wheat products. This study selected suitable colored wheat varieties for local conditions, compared different agronomic traits and yields, and ultimately identified dwarfing genes that increase yield. These genes were then validated, providing theoretical support for the breeding of high-yield colored wheat. This research aimed to provide valuable insights for the development of new varieties of colored wheat resources. Consequently, this study provides theoretical support for breeding high-yielding colored wheat and aims to offer valuable insights and guidance for the development of new varieties within the colored wheat resources. This endeavor is expected to inject new momentum and direction into the future development of the colored wheat breeding field.

Different yield components can lead to variations in yield under different environmental conditions. The spike number, grain number per spike, and thousand-grain weight determine the individual plant yield of colored wheat and are crucial yield components. Plant height and spike length are related to the lodging resistance of colored wheat and influence its overall yield. Therefore, to breed high-yielding, stable, and high-quality colored wheat varieties, it is essential to investigate these yield components and conduct in-depth analyses. Currently, there are numerous reports on the contributions of the three yield components to grain yield. However, due to variations in the use of varieties, sample sizes, and experimental conditions, the conclusions of previous research are inconsistent. Under specific conditions, the thousand-grain weight [7,8,9], grain number per spike [10], or spike number per unit area may all become the major contributors to yield. Furthermore, for some varieties, plant height is the primary determining factor for yield [11]. The deployment of Reduced height (*Rht*) semi-dwarfing genes (*Rht-B1* and *Rht-D1*) was a crucial component of the ‘Green Revolution’ for improving yield potential due to an increased harvest index and lodging resistance. Due to these factors, more than 70% of wheat cultivars grown globally nowadays incorporate at least one of these semi-dwarfing genes [12].

The genes controlling plant height include the *Rht* series of genes, each of which has a different impact on wheat plant height. Among them, the most widely used dwarf genes in wheat dwarfing breeding are *Rht-B1b* and *Rht-D1b* from the Agronomy 10 wheat cultivar, as well as *Rht* 8 from Japanese red wheat (AKAK-OMUGi) [13]. To further clarify the relationship between colored wheat traits and yield, this study utilized structural equation modeling, which is sometimes referred to as covariance structure analysis, in addition to covariance structure modeling and the analysis of covariance structures [14], as a multivariate analysis method. Initially developed for use in the social sciences, structural equation modeling has also been applied to address complex issues in ecology [15].

Although more than 20 dwarfing genes have been documented in wheat, only a few genes are presently utilized in wheat breeding. More loci or genes that regulate plant height without influencing yield components need to be investigated further. In this study, we identified the *Rht1*, *Rht-D1*, and *Rht8* genes that influence plant height. This finding holds significant implications for enhancing wheat yield. This study employed statistical methods to screen 72 varieties of colored wheat in China, selecting those suitable for local conditions. The selected varieties were further analyzed to determine their agronomic traits, and three dwarfing genes controlling plant height, namely *Rht1*, *Rht-D1*, and *Rht8*, were validated. This research aimed to provide valuable insights for the development of new varieties of colored wheat resources.

## 2. Materials and Methods

### 2.1. Experimental Materials

**Figure 1:**
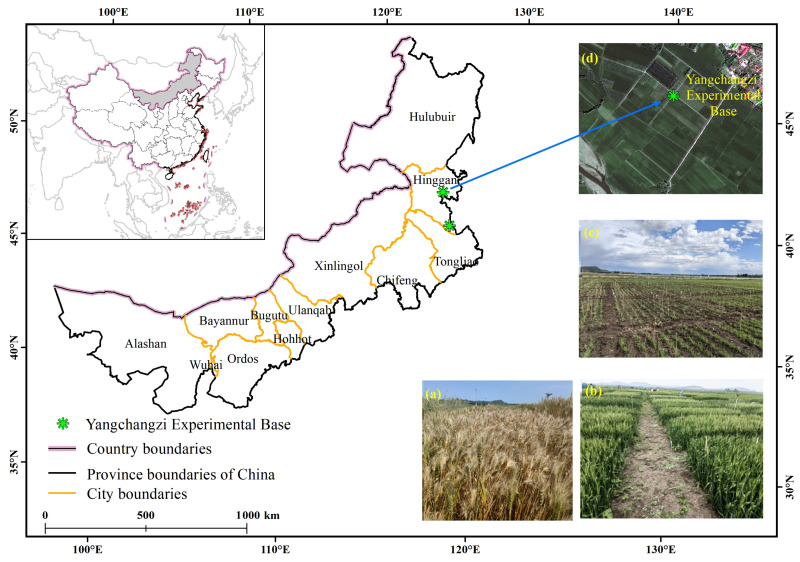
Yangchangzi Experimental Base of the Hinggan League Agricultural and Animal Husbandry Technology Extension Center (Yangchangzi village, Yilelite Town, Ulan Hot City, Hinggan League, Inner Mongolia). (**a**) Photographs illustrating the maturity stage of wheat; (**b**) photographs depicting the wheat flowering stage; (**c**) photographs illustrating the emergence stage of wheat seedlings; (**d**) Yang chang zi Experimental Base.

The experiment was conducted at the Yangchangzi Experimental Base (46°05′ N, 227°03′ E) of the Hinggan League Agricultural and Animal Husbandry Technology Extension Center during 2022–2023 (Figure 1). A total of 72 germplasm resources were utilized in this study (Table A1). These materials are all genetically stable varieties bred by various breeding units. The experimental plots were designed, managed in the field, and characterized based on the new standards of the “Technical Regulations for Regional Trials of Crop Varieties (Wheat)” issued by the Ministry of Agriculture. A total of six traits were investigated, including plant height, spike length, flag leaf length, flag leaf width, stem thickness, and yield.

### 2.2. Data Analysis

Data statistical analysis was performed using SPSS 20.0 software, and the “R package” was utilized for structural equation modeling. The entropy method was employed to screen out varieties. For index measurement, in each repetition, 10 plants were randomly harvested, and the parameters of plant height, ear length, flag leaf length, flag leaf width, stem thickness, and yield were statistically analyzed.

#### 2.2.1. Initial Structural Equation Model

**Figure 2:**
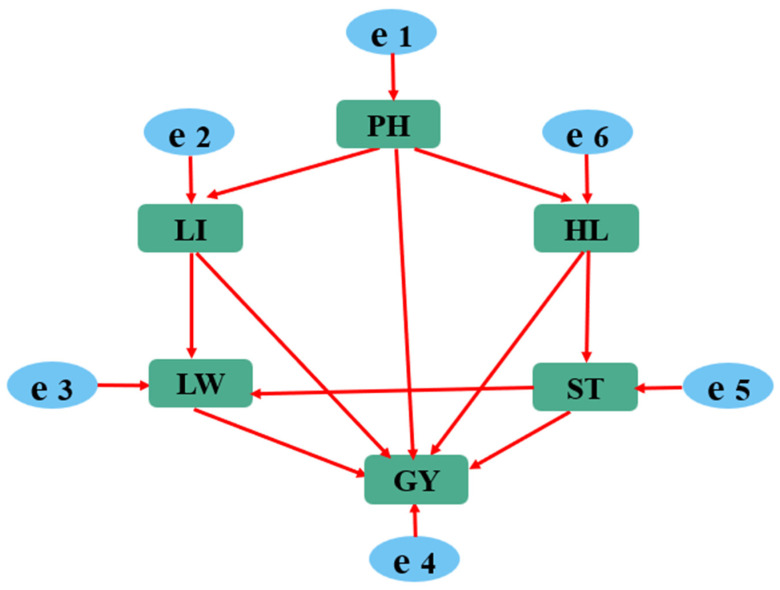
Initial modeling of the relationship between yield and agronomic traits in colored wheat. The initial model contains 16 relationships between parameters, which are shown by arrows, where e1 through e6 indicate errors. GY: grain yield; LI: leaf length; LW: leaf width; HL: head length; ST: stem thickness; PH: plant height.

A structural equation model is an a priori model [15], requiring the establishment of hypothesized paths based on practical experience and theory. Subsequently, through structural equation modeling, the direct and indirect relationships among various factors are analyzed, validated, and optimized. Ultimately, the rational causal relationships among the factors are determined [16]. The initial model for this study is illustrated in Figure 2. Based on the literature [16] and years of breeding experience, the initial model assumes that plant height, flag leaf length, flag leaf width, ear length, and stem thickness have direct effects on yield. Therefore, it was hypothesized that greater plant height leads to more tillers, resulting in a greater number of ears. After setting up the initial model, the estimation of 16 relationships between parameters was required (Figure 2), which was fewer than the total number of equations that could be established [6 (6 + 1)/2 = 21]. Thus, the model was identifiable.

#### 2.2.2. Model Verification

The overall evaluation of the model was conducted using the χ^2^ test [16], where a smaller χ^2^ value indicates a better fit of the model. If *p* > 0.05, it indicates that the model does not omit important parameters. In this study, there were a total of 432 (72 × 6) data points, meeting the minimum recommended sample size for the χ^2^ test in structural equation modeling, which is at least five times the number of model parameters [17]. The fit of the model was further assessed using the root mean square error of approximation (RMSEA) and the Akaike information criterion (AIC) [18]. For RMSEA, a value less than 0.05 indicates an excellent fit; 0.05 ≤ RMSEA < 0.08 suggests a good fit; 0.08 ≤ RMSEA < 0.10 indicates an acceptable fit; and RMSEA ≥ 0.10 suggests a poor fit. For the AIC, a lower value indicates a better fit of the model.

#### 2.2.3. Entropy Method and Indicator Selection

Entropy method: The entropy method is a technique for objectively assigning weights to indicators based on the magnitude of their information entropy. A smaller value of information entropy indicates a greater degree of dispersion for the indicator, implying more information content and therefore a higher weight. To overcome the limitations of the entropy method in dealing only with cross-sectional data and not being able to compare different varieties, this study adopted the panel entropy method [19].

### 2.3. Transcriptome Sequencing and Real-Time Fluorescence Quantitative Polymerase Chain Reaction (RT-qPCR) Validation of Candidate Genes

Based on the analysis of the above agronomic traits, 10 varieties of colored wheat were selected (Table A2). The total RNA was extracted using the TRizol method, and the transcriptional sequencing was performed by Beijing Genomics Company (BGI) (the instruments are listed in Table A3). The DNBSEQ high-throughput platform was utilized for sequencing, followed by subsequent bioinformatics analysis.

First, sample detection involves taking a certain amount of RNA samples, denaturing their secondary structures at an appropriate temperature, and enriching mRNA using oligo(dT) magnetic beads. In the subsequent step, mRNA obtained from the previous step is subjected to fragmentation by adding a disruption reagent and reacting at an appropriate temperature for a certain duration, resulting in fragmented mRNA. The fragmented mRNA is then subjected to a one-step synthesis reaction system previously prepared, and a reaction program is set up to synthesize single-stranded cDNA. A two-step synthesis reaction system (containing dUTP) is prepared, and a reaction program is set up to synthesize double-stranded cDNA. A reaction system is prepared, and a reaction program is set up to repair the ends of the double-stranded cDNA and add an A base at the 3′ end. A reaction system for adapter ligation is prepared, and a reaction program is set up to ligate the adapters to the cDNA.

Next, a PCR reaction system is prepared, and a reaction program is set up to amplify the products. According to the requirements of the product, appropriate detection methods are selected for library quality control. After denaturing the PCR products into single strands, a ligation reaction system is prepared, and a reaction program is set up to obtain single-stranded circular products, digesting the linear DNA molecules that are not circularized. The single-stranded circular DNA molecules undergo rolling circle amplification, forming DNA nanoballs (DNBs) containing multiple copies. The obtained DNBs are loaded into the mesh-like pores on a high-density DNA nanochip, and sequencing is performed using combinatorial probe–anchor synthesis (cPAS) technology. Each material had three biological replicates. The expression levels of three candidate dwarfing genes, *Rht1*, *Rht-D1*, and *Rht8*, were obtained through transcriptome analysis (specific method, the 2^−ΔΔCT^ (CT, cycle threshold) method was employed to calculate the relative expression levels of the candidate genes in different varieties), and a heat map was generated. The expression characteristics of different materials were determined using the RT-qPCR method. The primer sequences are shown in Table A2.

#### 2.3.1. RNA Extraction

First, 1 mL of Trizol was added to a tissue sample that was approximately the size of a green bean, homogenized for 1 min using a Tissue Ruptor, and then allowed to stand at room temperature for 10 min. Then, 200 μL of chloroform was added, thoroughly mixed, and centrifuged at 15,000 rpm for 5–7 min. After this, the supernatant was transferred to a 1.5 mL Eppendorf tube, 600 μL of chloroform was added and mixed in, and the mixture was centrifuged at 15,000 rpm for 5 min.

Next, the supernatant was transferred to a 1.5 mL Eppendorf tube, 500 μL of isopropanol was added and mixed, and the mixture was then centrifuged at 15,000 rpm for 10 min. The supernatant was then discarded, and the remaining RNA pellet was washed with 1 mL of 75% ethanol before being centrifuged at high speed for 5 min. Subsequently, the supernatant was again discarded, and the RNA pellet was air dried for 2–3 min, after which the dried RNA pellet was washed in RNA-free water. Finally, 1 μL of the total RNA was taken for OD260 measurement and quantification (for reagents, assay kits, and instruments, see Table A4 and Table A5).

#### 2.3.2. Reverse Transcription

The following reagents were sequentially added to a 200 μL PCR tube, totaling 12 μL; (10 − *x*) μL of DEPC (Diethylpyrocarbonate) water; 2 μL of Random primers/Oligo dT (50 pM/μL); and *x* μL of RNA (2 μg). The tube was then incubated at 65 °C for 5 min in a PCR machine, followed by immediate transfer to an ice bath and centrifugation at a high speed (above 5000× *g*) for 5 s. Subsequently, the following reagents were added to the PCR tube in the specified order, resulting in a total volume of 20 μL after their addition: 1 μL of RiboLock RNase Inhibitor; 4 μL of 5×Reaction Buffer; 2 μL of dNTP (Deoxyribonucleotide Triphosphate) Mix; and 1 μL of RevertAid Reverse Transcriptase.

The ingredients in the tube were mixed well, after which they were incubated at 25 °C for 5 min and then at 42 °C for 60 min (for samples with a high GC (gas chromatography) content, the temperature could be increased to 45 °C). Then, the tube was incubated at 70 °C for 5 min and subsequently centrifuged at a high speed (above 5000× *g*) for 5 s. The contents were then stored at −20 °C, but for longer storage, they could be stored at −80 °C.

#### 2.3.3. Fluorescent Quantitative RT-qPCR Amplification

Sequence and primer design: sequences were referenced from the Gene Bank database for each target gene, and primers were designed using the National Center for Biotechnology Information (NCBI) Primer-Blast and synthesized by Suzhou Jinweizhi (Table A2).

RT-qPCR amplification, for sequence and primer design, sequences referred to the sequence of the target gene in the GenBank database, while primers were designed using the NCBI’s Primer-BLAST and synthesized by Suzhou Jinwei Zhi (Table A2).

The PCR reaction system (10 μL) consisted of 1.5 μL of H_2_O, 5 μL of 2 × SYBR GREEN PCR mix, 1 μL of Primer (10 μM), and 2.5 μL of Template (cDNA). Reaction conditions were as follows: initial denaturation at 95 °C for 2 min, followed by denaturation at 95 °C for 5 s, annealing at 60 °C for 10 s (for 45 cycles), and a melt curve analysis.

Specific method, the 2^−ΔΔCT^ (CT, Cycle Threshold) method was employed to calculate the relative expression levels of the candidate genes in different varieties. Total RNA extraction: the total RNA was extracted using the Trizol method and quantified.

## 3. Results and Analysis

### 3.1. Basic Statistical Characteristics of Colored Wheat

The coefficients of variation for plant height, flag leaf length, flag leaf width, ear length, stem thickness, and yield of colored wheat ranged from 2.86% to 56.14%, with yield exhibiting the highest variability at 56.14%. As shown in Table 1, the highest yield was 5744.1 kg hm^−2^ for variety 70, while the lowest yield was 382.05 kg hm^−2^, resulting in a range of 2709.15 kg hm^−2^ (Table 1).

### 3.2. Correlation Analysis of Main Agronomic Traits and Yield in Colored Wheat

A correlation analysis was performed to determine the correlations between the main agronomic traits and yield in colored wheat. The correlation between plant height and flag leaf length, as well as ear length, reached a highly significant level, with correlation coefficients of 0.306 and 0.404, respectively. The correlation between plant height and yield was significant, with a coefficient of 0.257. The correlation between flag leaf length and ear length was highly significant, with a coefficient of 0.334. Yield showed a significant correlation with ear length, with a coefficient of 0.45 (Table 2).

### 3.3. Using Structural Equation Modeling to Analyze the Relationship between the Main Agronomic Traits and Yield in Colored Wheat

The fit results of the initial model showed that χ^2^ was 5.626 with four degrees of freedom (df), resulting in a χ^2^/df ratio of 1.4065 and *p* < 0.05. However, both the root mean square residual (RMR) and RMSEA were greater than 0.05 (Table 3). Therefore, the model required revision. During the revision, the significant path coefficients were retained and the non-significant path coefficients were removed. Structural equation models, as a priori models, allow for the retention of some statistically insignificant paths with biological significance based on the actual growth of crops. In the final revised model, the path from stem thickness to flag leaf width was eliminated. The revised model parameters are shown in Table 3, with a *p*-value of 0.025 and both RMR and RMSEA values of less than 0.05.

**Figure 3:**
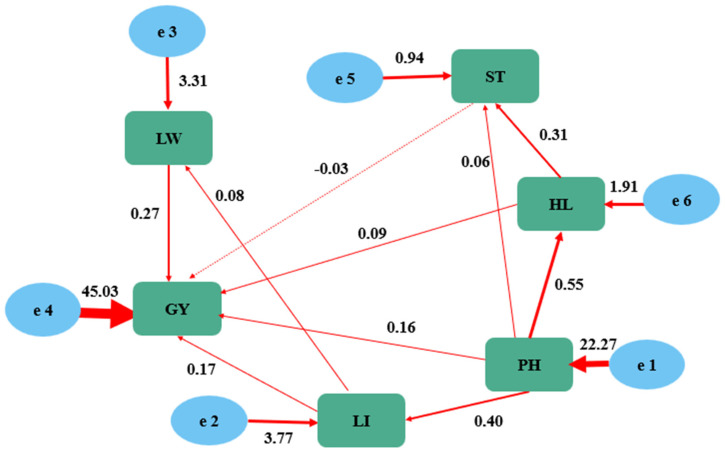
Effects of agronomic traits on the yield of colored wheat and relationships among agronomic traits as determined by structural equation modeling. Note: Red lines show significant paths, with greater line thickness reflecting the greater size of the absolute path coefficient. The dashed line shows that the path is not significant, but this path needs to be conserved based on the physiological process of colored wheat. The values beside the lines are standard path coefficients, and the values beside the rectangles and ovals are determination coefficients.

In the final structural equation model established, all agronomic traits had positive direct effects on yield. Among them, plant height, ear length, and flag leaf width had significant impacts on yield, with path coefficients of 0.55, 0.40, and 0.27, respectively. The path coefficient of flag leaf length on yield was 0.17, while the influence of stem thickness on yield was relatively small. Each trait also exerted an effect on the final yield through their mutual effects (Figure 3). This indicates that random variations in the environment where colored wheat is grown have a significant impact on each agronomic trait, and once these trait characteristics are determined, the final yield is essentially determined.

### 3.4. Using the Entropy Method to Screen Colored Wheat Seed Resources

Structural equation modeling was used to determine the impacts of agronomic traits on yield. The next step involved using the entropy method to screen locally suitable varieties of colored wheat based on these agronomic traits (Table 4 and Table A2), facilitating promotion and demonstration planting for farmers and herders. The agronomic trait panel data for 72 colored wheat germplasm resources in the 2022–2023 trial base, including the plant height, flag leaf length, flag leaf width, main spike length, main spike stem thickness, and yield per hectare, represented attributes in six dimensions. The entropy method was utilized to calculate the weights of the six indicators and the final overall scores. The entropy method comprehensively evaluated the agronomic traits of the 72 wheat varieties, resulting in the calculation of composite indices for each variety. Finally, 10 colored wheat varieties that were suitable for the local conditions were selected (Table 4). The specific classification weights are shown in Table 5.

### 3.5. Screening for Dwarfing Genes in Colored Wheat

**Figure 4:**
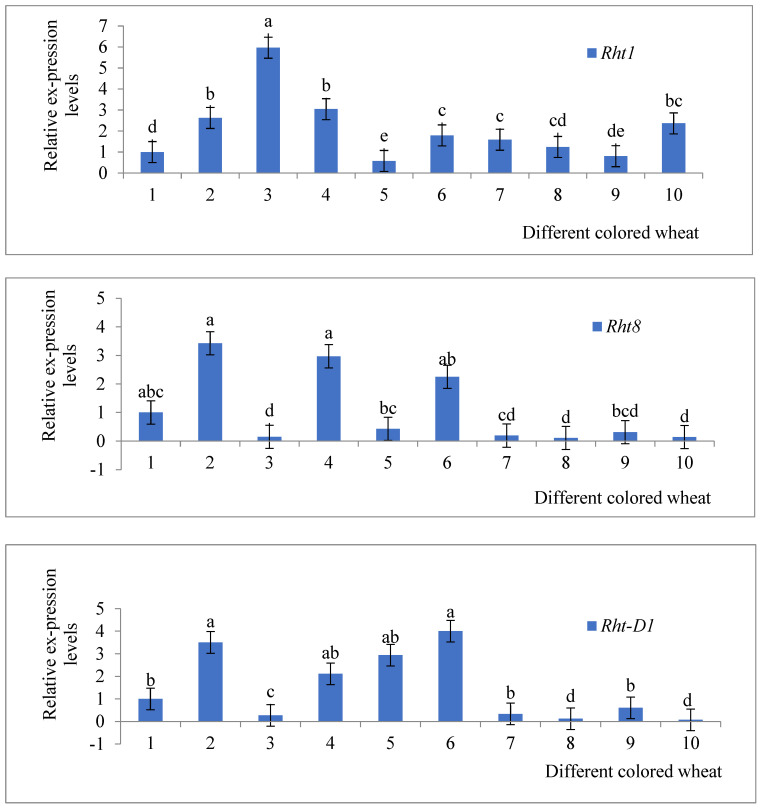
RT-qPCR results of three dwarfing genes in colored wheat. RT-qPCR: real-time fluorescence quantitative polymerase chain reaction. Values are the mean ± SE of three biological replicates, and different letters indicate significant differences among different samples using Duncan’s test (*p* < 0.05).

**Figure 5:**
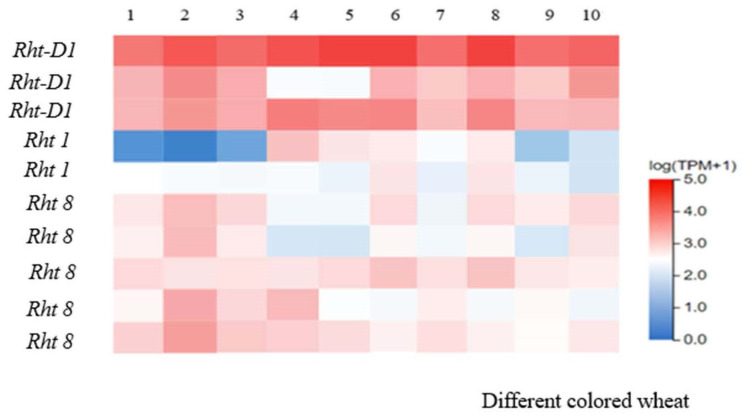
Heatmaps depicting the relative expression of the studied reduced dwarfing genes in different colored wheat lines. The heatmaps were generated from the transcriptome sequencing of colored wheat leaf blades. The transcriptional sequencing was performed by Beijing Genomics Company (BGI). The DNBSEQ high-throughput platform was utilized for sequencing. The level of normalized gene expression from high to low is indicated by the color scheme from red to white to blue. Numbers 1~10: different colored wheat. dwarfing genes: *Rht1*, *Rht8*, and *Rht-D1*.

This research employed transcriptome sequencing (conducted by BGI Genomics) and RT-qPCR validation (sequence and primer design in Table A2). The estimated expression patterns for each biosynthetic gene were validated through RT-qPCR (Figure 4). Similar expression patterns were observed in the transcriptome data for randomly selected biosynthetic genes. Notably, *Rht1*, *Rht8*, and *Rht-D1* showed high expression levels in colored wheat. The RT-qPCR reports for 10 different colored wheat varieties indicated the presence of three dwarfing genes that control plant height: *Rht1*, *Rht-D1*, and *Rht8*. The clustering heatmap revealed that semi-dwarf genes, such as *Rht-D1* and *Rht8*, exhibited consistent trends in both plant height variation and the RT-qPCR reports (Figure 5), while the trend for *Rht1* gene expression differed slightly. Analyzing the results from a yield perspective (Table 4), variety 6, characterized by dwarf stature, showed high expression in both the clustering heatmap (Figure 5) and RT-qPCR reports and ranked second in grain yield. This suggests that dwarf varieties not only resist lodging but also have high yields.

## 4. Discussion

Dwarfing genes have been a major driver of improved adaptation and performance with breeding and domestication across all the major cereals [20]. In colored wheat, the identification and deployment of genes for semi-dwarf stature have promoted the commercial release and global adoption of wheat cultivars with greater yield potential and stability [21]. Selection for reduced crop height remains a key objective of colored wheat breeding programs worldwide, owing to semi-dwarfs being less prone to lodging and producing greater amounts of grain to increase their harvest index and crop yields [22]. In this study, three dwarfing genes, *Rht1*, *Rht-D1*, and *Rht8*, were identified in 10 colored wheat varieties (Figure 4). The RT-qPCR markers for each dwarfing gene were specific, allowing for gene differentiation. Sequencing of different wheat varieties for the *Rht1*, *Rht-D1*, and *Rht8* alleles was used to create a heatmap, which indicated that semi-dwarfing gene varieties such as *Rht1* and *Rht8* roughly corresponded with the changes in plant height reported by RT-qPCR (Figure 4). However, the trend of the *Rht1* gene differed somewhat from the RT-qPCR reports, possibly due to environmental variations. Molecular detection and transcriptome sequencing findings were generally consistent with the RT-qPCR results. Sequence-tagged site markers based on RT-qPCR can be used for the identification of the *Rht1*, *Rht-D1*, and *Rht8* genes in colored wheat varieties and for screening in breeding progeny.

The discovery of *Rht* genes played a crucial role in the development of modern high-yielding wheat varieties due to their capacity to allocate more energy to grain production [23]. With regards to the impact of the *Rht1* and *Rht8* mutations on plant height, the effects observed were consistent with those reported by Flintham et al. [24]. Regarding leaf morphology traits, we reported an increasing trend in leaf length and width caused by dwarfing allele genes. This trend can be explained by an increased allocation of photosynthetic products in developing leaves [25]. Additionally, higher length fertility led to a higher harvest index, supporting the views on semi-dwarfing genes *Rht1* and *Rht8*.

The results of structural equation modeling indicated that the effects of each agronomic trait on yield were positive. Among them, plant height, ear length, and flag leaf width had significant impacts on yield. Our study suggests that the random variations in the environment where colored wheat is grown have a significant impact on each agronomic trait. However, once these agronomic trait characteristics are determined, the final yield can be essentially predicted. Plant height, as a crucial agronomic trait in cereal crops, has a significant impact on crop yield potential and stability. Studies have shown that reducing plant height can enhance a plant’s lodging resistance [26,27]. In the 1960s, dwarfing genes were first applied in wheat breeding, triggering the first “Green Revolution” [28]. The positive role of the Green Revolution gene *Rht1* that led to higher grain yield potential has been extensively reported worldwide [24]. In agreement with our results, Sherman et al. [29] and Tang et al. [30] indicate that the semi-dwarfed plants carrying these genes are associated with an increase in productive tillers and grains per spike, leading to higher GN and grain yield.

To date, a total of 25 dwarfing genes (*Rht* genes) have been identified in wheat, and each *Rht* gene has different effects on wheat plant height. Among them, the most widely used dwarfing genes in wheat breeding for reduced plant height are *Rht-D1b* from Norin 10 and *Rht8* from Japanese red wheat (AKAK-OMUGi) [31], respectively. Studies have shown that when the *Rht-D1b* gene is present alone, the dwarfing effect is approximately 24%, and when both genes are present simultaneously, there is a cumulative effect with a dwarfing effect of nearly 60% [32,33]. The dwarfing effect of *Rht8* is 11% [34], indicating a weaker dwarfing ability compared to *Rht-D1b* [35]. This is consistent with the high yield of the cultivars carrying dwarf genes in this study. Variety 6 is a short-stemmed variety with high expression in cluster heatmaps and RT-qPCR reports, ranking first in grain yield. This indicates that short-stemmed varieties can not only resist lodging but also have high yields.

## 5. Conclusions

This study identified that each agronomic trait has a positive direct effect on yield. Additionally, each trait also influences the final yield through its intercorrelated effects. This suggests that random environmental variations during the production of colored wheat significantly impact various agronomic traits. Regarding leaf morphology traits, we observed an increasing trend in leaf length and width induced by dwarfing allele genes. This phenomenon can be attributed to an enhanced allocation of photosynthetic products in developing leaves. Analyzing the results from a yield perspective reveals that short-stemmed varieties not only exhibit lodging resistance but also demonstrate an increasing trend in leaf length and width, contributing to higher yields. Therefore, researching dwarfing genes is of great significance, and our study provides valuable insights for the development of new high-yielding varieties of colored wheat. They hold potential for local cultivation and promotion.

## Figures and Tables

**Table 1:** The coefficient of variation for various agronomic traits of different colored wheat varieties.

Traits	Minimum	Maximum	Mean	SD	CV (%)
Plant height (cm)	42	110.33	71.12	16.49	23.19
Leaf length (cm)	13.67	33.00	24.83	3.77	15.20
Leaf width (cm)	0.97	2.30	1.56	0.28	17.84
Head length (cm)	5.33	13.83	9.01	1.91	21.18
Stem thickness (mm)	30.30	35.14	32.82	0.94	2.86
Yield (kg hm^−2^)	382.05	5744.1	2709.15	101.40	56.14

Note: SD, standard deviation; CV, coefficient of variation.

**Table 2:** Correlation analysis of agronomic characteristics of colored wheat.

No.	Plant Height (cm)	Leaf Length (cm)	Leaf Width (cm)	Head Length (cm)	Stem Thickness (mm)	Yield (kg hm^−2^)
Plant height (cm)	1	0.306 **	−0.038	0.404 **	0.002	0.257 *
Leaf length (cm)		1	0.123	0.334 **	−0.084	0.184
Leaf width (cm)			1	−0.100	−0.261 *	−0.097
Head length (cm)				1	−0.209	0.45 *
Stem thickness (mm)					1	0.100
Yield (kg hm^−2^)						1

* *p* < 0.05; ** *p* < 0.01.

**Table 3:** Initial and final fit indices of structural equation models.

Model	χ^2^ (df)	χ^2^/df	*p*	RMR	RMSEA	AIC	GFI	NFI	CFI
Initial model	5.626 (4)	1.4065	0.000	0.051	0.082	819.022	0.968	0.913	0.967
Final model	12.802 (5)	2.5604	0.025	0.021	0.041	824.197	0.933	0.803	0.844

Note: χ^2^: Chi-square; df: degree of freedom; χ^2^/df: square of mean; *p*: probability value; RMR: root mean square residual; RMSEA: root mean square error of approximation; AIC: Akaike information criterion; GFI: goodness-of-fit index; NFI: normal fit index; CFI: comparative fit index.

**Table 4:** A list of the top 10 ranked wheat varieties determined by the entropy method using 72 colored wheat.

No.	Variety Serial Number	Color	Plant Height (cm)	Leaf Length (cm)	Leaf Width (cm)	Head Length (cm)	Stem Thickness (mm)	Yield (kg hm^−2^)	Composite Index
1	70	Purple–Black	106.67 ± 1.65	26.83 ± 8.36	1.70 ± 3.25	11.67 ± 5.33	33.50 ± 1.11	5571.90 ± 0.77	87.15
2	6	Purple	42.67 ± 0.29	24.00 ± 0.84	1.80 ± 0.11	9.67 ± 1.20	32.93 ± 4.87	5744.10 ± 0.20	81.32
3	11	Purple–Black	96.00 ± 0.09	28.83 ± 3.0	1.73 ± 0.26	13.83 ± 1.2	33.57 ± 0.87	5088 ± 2.29	79.91
4	3	Purple	72.33 ± 0.16	28.00 ± 0.98	1.57 ± 3.06	10.50 ± 0.42	33.60 ± 3.22	5170.35 ± 0.20	76.64
5	1	Black	68.67 ± 0.05	27.50 ± 0.44	2.03 ± 0.55	11.33 ± 0.22	33.87 ± 5.21	4839.45 ± 3.22	72.42
6	5	Black	58.33 ± 0.55	19.67 ± 0.05	1.80 ± 0.11	6.67 ± 2.0	32.83 ± 5.72	5001.30 ± 0.98	71.89
7	2	Purple–Black	60.33 ± 0.00	31.33 ± 0.55	1.60 ± 0.05	11.00 ± 3.22	33.89 ± 1.11	4472.55 ± 0.20	71.66
8	8	Purple–Black	62.67 ± 0.95	24.17 ± 1.06	1.97 ± 2.01	10.83 ± 3.21	33.67 ± 0.22	4746.60 ± 4.21	70.15
9	10	Purple	63.33 ± 0.01	25.00 ± 1.06	1.80 ± 0.06	10.67 ± 3.25	33.36 ± 2.28	4664.85 ± 0.44	69.21
10	4	Purple	91.00 ± 0.01	13.67 ± 0.20	1.60 ± 0.06	6.83 ± 0.06	33.12 ± 6.24	4768.65 ± 0.87	66.28

**Table 5:** The weights of the top 10 ranked wheat varieties determined by the entropy method using 72 color-coded samples.

	Weight		Weight
Plant height	0.16532		
Leaf area index	0.55325	Leaf length	0.05961
		Leaf width	0.94039
Head	0.10456	Head length	0.63697
		Stem thickness	0.36303
Yield	0.17686		

## Data Availability

The data are reliable and usable.

## Appendix A

**Table A1:** The different colors and varieties of resources for colored wheat.

No.	Resource	Color	Source	No.	Resource	Color	Source
1	KY2022-1	Purple	Chinese Inner Mongolia Academy of Agriculture and Animal Husbandry Sciences	37	ZNY2022-24	White	Chinese Academy of Sciences
2	KY2022-2	Purple	Chinese Inner Mongolia Academy of Agriculture and Animal Husbandry Sciences	38	ZNY2022-25	White	Chinese Academy of Sciences
3	LY2022-1	Purple	Chinese Inner Mongolia Yuanlu Company	39	ZNY2022-26	White	Chinese Academy of Sciences
4	QY2022-1	Purple	Northwest A&F University	40	ZNY2022-27	White	Chinese Academy of Sciences
5	QY2022-2	Blue	Northwest A&F University	41	ZNY2022-28	White	Chinese Academy of Sciences
6	QY2022-3	Purple	Northwest A&F University	42	ZNY2022-29	White	Chinese Academy of Sciences
7	QY2022-4	Purple	Northwest A&F University	43	ZNY2022-30	White	Chinese Academy of Sciences
8	QY2022-5	Purple	Northwest A&F University	44	ZNY2022-31	White	Chinese Academy of Sciences
9	QY2022-6	White	Northwest A&F University	45	ZNY2022-32	White	Chinese Academy of Sciences
10	NXY2022-1	Purple	Ningxia University	46	ZNY2022-33	White	Chinese Academy of Sciences
11	MDY2022-1	Purple	Inner Mongolia	47	ZNY2022-34	White	Chinese Academy of Sciences
12	ZKYY2022-1	Purple	Chinese Academy of Sciences	48	ZNY2022-35	White	Chinese Academy of Sciences
13	ZKYY2022-2	Purple	Chinese Academy of Sciences	49	ZNY2022-36	White	Chinese Academy of Sciences
14	ZNY2022-1	Purple	Chinese Academy of Sciences	50	ZNY2022-37	White	Chinese Academy of Sciences
15	ZNY2022-2	Purple	Chinese Academy of Sciences	51	ZNY2022-38	White	Chinese Academy of Sciences
16	ZNY2022-3	Purple	Chinese Academy of Sciences	52	ZNY2022-39	White	Chinese Academy of Sciences
17	ZNY2022-4	Purple	Chinese Academy of Sciences	53	ZNY2022-40	White	Chinese Academy of Sciences
18	ZNY2022-5	Purple	Chinese Academy of Sciences	54	ZNY2022-41	White	Chinese Academy of Sciences
19	ZNY2022-6	Purple	Chinese Academy of Sciences	55	ZNY2022-42	White	Chinese Academy of Sciences
20	ZNY2022-7	Purple	Chinese Academy of Sciences	56	ZNY2022-43	White	Chinese Academy of Sciences
21	ZNY2022-8	Purple	Chinese Academy of Sciences	57	ZNY2022-44	White	Chinese Academy of Sciences
22	ZNY2022-9	Purple	Chinese Academy of Sciences	58	ZNY2022-45	White	Chinese Academy of Sciences
23	ZNY2022-10	Purple	Chinese Academy of Sciences	59	ZNY2022-46	White	Inner Mongolia Huhe hot
24	ZNY2022-11	Purple	Chinese Academy of Sciences	60	ZNY2022-47	Purple	Inner Mongolia Academy of Agricultural Sciences
25	ZNY2022-12	Purple	Chinese Academy of Sciences	61	ZNY2022-48	Purple	Inner Mongolia Academy of Agricultural Sciences
26	ZNY2022-13	Purple	Chinese Academy of Sciences	62	ZNY2022-49	Purple	Inner Mongolia Academy of Agricultural Sciences
27	ZNY2022-14	Purple	Chinese Academy of Sciences	63	ZNY2022-50	Purple	Inner Mongolia Academy of Agricultural Sciences
28	ZNY2022-15	Purple	Chinese Academy of Sciences	64	ZNY2022-51	Purple	Inner Mongolia Academy of Agricultural Sciences
29	ZNY2022-16	Purple	Chinese Academy of Sciences	65	ZNY2022-52	Purple	Inner Mongolia Academy of Agricultural Sciences
30	ZNY2022-17	Purple	Chinese Academy of Sciences	66	ZNY2022-53	Purple	Inner Mongolia Academy of Agricultural Sciences
31	ZNY2022-18	White	Chinese Academy of Sciences	67	ZNY2022-54	Purple	Inner Mongolia Academy of Agricultural Sciences
32	ZNY2022-19	White	Chinese Academy of Sciences	68	ZNY2022-55	Purple	Inner Mongolia Academy of Agricultural Sciences
33	ZNY2022-20	White	Chinese Academy of Sciences	69	ZNY2022-56	Purple	Inner Mongolia Academy of Agricultural Sciences
34	ZNY2022-21	White	Chinese Academy of Sciences	70	ZNY2022-57	Purple	Inner Mongolia Ulanhot
35	ZNY2022-22	White	Chinese Academy of Sciences	71	ZNY2022-58	Purple	Inner Mongolia Shangkuli Farm Hulunbuir
36	ZNY2022-23	White	Chinese Academy of Sciences	72	ZNY2022-59	Purple	Inner Mongolia Shangkuli Farm Hulunbuir

**Table A2:** The sequences and primer designs employed in the RT-qPCR experiments on colored wheat.

NM	Amplification Length (bp)	Primer Name	Sequence (5′–3′)
FN649763.1	114 bp	Triticum *RHT1* F-primer	cgccatgttcgattctctgg
		Triticum *RHT1* R-primer	tacacctcggacatgacctg
OL958546.1	116 bp	Triticum *Rht8* F-primer	gactctgcgtgatctcgaga
		Triticum *Rht8* R-primer	cgttgttgaagagtgggagc
KC614643.1	60 bp	Triticum *Rht-D1* F-primer	tccgaggacaagatgatggt
		Triticum *Rht-D1* R-primer	cagctcgtccacctcctc

Note: The sequences are referenced from the GenBank database for each target gene. Primers were designed using NCBI Primer-BLAST and synthesized by Suzhou Jinweizhi.

**Table A3:** The reagents and laboratory equipment used in the transcriptome sequencing experiment of colored wheat.

Name	Reagents			Instrumentation and Equipment		
	The Names of the Reagents	Brand	Catalog Number	Instrument Name	Brand	Model
Introduction to the Experimental Procedure for Plant Tissue RNA Extraction	TRIzol Reagent	Invitrogen (A biotechnology company in the United States.)	15596026	Fully automated sample rapid grinder	Shanghai Jingxin Technology	JXFSTPRP-48
	Trichloromethane	Xilong Chemicals (A chemical company in China.)	AR500ML	Centrifuge	Eppendorf	5417R
	Isopentanol	Xilong Chemicals (A chemical company in China.)	AR500ML	Pipette	Eppendorf	
	Isopropanol (analytical grade)	Xilong Chemicals (A chemical company in China.)	AR500ML	Vortex mixer	Kylin-Bell	QL-901
	Anhydrous ethanol (analytical grade)	Xilong Chemicals	72188-01			
	DEPC-treated water	AMBION	AM9922			
Introduction to the Procedure for Chain-Specific mRNA Library Preparation (DNBSEQ)	DNF-471 STANDARD SENSITIVITY RNA ANALYSIS KIT	AATI	DNF-471-0500	Fragment Analyzer	Agilent	5300
	BGI Optimal series dual-module mRNA library construction kit	BGI	LR00R96	Microcentrifuge	Baygene	BG Qspin7000
	BGI Plug-In Adapter Kit	BGI	LA00R04	Vortex mixer	Kylin-Bell	QL-901
	DNA separation magnetic beads	BGI	LB00V60	Automated pipetting workstation	MGI	MGISP-960
	Qubit^®^ ssDNA Assay Kit	Invitrogen	Q10212	PCR instrument	ABI APPIED BIOSYSTEMS	9700
	MGISEQ-2000RS High-throughput sequencing reagent kit (FCL PE100)	MGI	1000012554	Pipette	Eppendorf	
	MGISEQ-2000RS High-throughput sequencing reagent kit(FCL PE150)	MGI	1000012555	Qubit	Thermo Fisher	Q33216
	DNBSEQ-T7RS High-throughput sequencing reagent kit (FCL PE100)	MGI	1000028455	Gene sequencer	MGI	MGISEQ-2000

**Table A4:** The reagents and assay kits used in the RT-qPCR experiments on colored wheat.

Reagents and Reagent Kits	Source
TRIzol™ Reagent Invitrogen™	Invitrogen 15596018 (A biotechnology company in the United States.)
RNase-Free Water	QIAGEN 129112 (A biotechnology company in the United States.)
First Strand cDNA synthesis Kit	Invitrogen K1622 (A biotechnology company in the United States.)
QuantityNova SYBR Green PCR Kit	QIAGEN 208052 (A biotechnology company in the United States.)
TBE	City of Shanghai Weiao Biological Technology Company Limited in China.
6*Loading buffer	City of Shanghai Weiao Biological Technology Company Limited in China.

**Table A5:** The experimental instruments used in the RT-qPCR experiments on colored wheat.

Instrument Name	Instrument Model	Manufacturer
Biological Safety Cabinet	BHC-1300	City of Suzhou Purification Equipment Company Limited in China.
Tissue Homogenizer	TL-2010S	City of Beijing Dinghaoyuan Technology Company Limited in China.
Tabletop High-Speed Centrifuge	Fresco21	Thermo Fisher Scientific, headquartered in Wilmington, MA, USA
Full Wavelength Microplate Reader	MutiskanTM GO	Thermo Fisher Scientific, headquartered in Wilmington, MA, USA
Bioanalyzer	BIO RAD POWER PAC 3000	BIO RAD, based in Hercules, CA, USA
Wide Mini-Sub Cell Horizontal Electrophoresis System	BIO RAD DNA SUB CELL	BIO RAD, based in Hercules, CA, USA
Gel Imaging System	GIS-1600	City of Shanghai Tianneng Technology Company Limited in China
Gradient PCR Instrument	Veriti DX	Thermo Fisher Scientific, headquar-tered in Wilmington, MA, USA
Fluorescent Quantitative PCR Instrument	Roche480II Real Time PCR System	Roche, headquartered in Basel, Switzerland
